# Rho A Regulates Epidermal Growth Factor-Induced Human Osteosarcoma MG63 Cell Migration

**DOI:** 10.3390/ijms19051437

**Published:** 2018-05-11

**Authors:** Jinyang Wang, Lei Zhang, Rongmei Qu, Lin Zhang, Wenhua Huang

**Affiliations:** 1 Department of Anatomy, Guangdong Provincial Key Laboratory of Tissue Construction and Detection, Southern Medical University, Guangzhou 510515, China; wangjy327@163.com (J.W.); qurongmei@163.com (R.Q.); 2 Department of Histology and Embryology, Southern Medical University, Guangzhou 510515, China; johnny399@126.com

**Keywords:** osteosarcoma, migration, stress fiber, Rho A

## Abstract

Osteosarcoma, the most common primary bone tumor, occurs most frequently in children and adolescents and has a 5-year survival rate, which is unsatisfactory. As epidermal growth factor receptor (EGFR) positively correlates with TNM (tumor-node-metastasis) stage in osteosarcoma, EGFR may play an important role in its progression. The purpose of this study was to explore potential mechanisms underlying this correlation. We found that EGF promotes MG63 cell migration and invasion as well as stress fiber formation via Rho A activation and that these effects can be reversed by inhibiting Rho A expression. In addition, molecules downstream of Rho A, including ROCK1, LIMK2, and Cofilin, are activated by EGF in MG63 cells, leading to actin stress fiber formation and cell migration. Moreover, inhibition of ROCK1, LIMK2, or Cofilin in MG63 cells using known inhibitors or short hairpin RNA (shRNA) prevents actin stress fiber formation and cell migration. Thus, we conclude that Rho A/ROCK1/LIMK2/Cofilin signaling mediates actin microfilament formation in MG63 cells upon EGFR activation. This novel pathway provides a promising target for preventing osteosarcoma progression and for treating this cancer.

## 1. Introduction

Osteosarcoma (OS) is the most common malignant bone-derived tumor and is particularly prevalent among children and adolescents. Although the long-term survival rate of OS patients has increased from 10% to nearly 80% within the last 25 years due to the use of neoadjuvant chemotherapy [1], survival rates have plateaued [2] and not risen over the last 15 years [3]. Furthermore, despite recent advances in effective treatments, including surgery and chemotherapy, the 5-year survival rate of OS patients with lung metastasis is less than 30% [4]. A better understanding of the molecular mechanisms involved in cancer progression is needed to reduce the financial burden and suffering of OS patients, to improve the treatment of OS, and to reduce the metastasis rate. Indeed, the mechanisms leading to OS metastasis remain unknown and represent an unmet challenge.

Cancer cell migration is a complex process that requires multiple steps and the participation of multiple signaling molecules. In general, these actions are controlled by a signaling cascade that is initiated by adhesion molecules (e.g., integrins) or growth factor receptors, such as the epidermal growth factor receptor (EGFR) [5]. Several studies have shown that EGFR is expressed in bone and soft tissue tumors and that its expression is positively correlated with TNM (tumor-node-metastasis) stage in OS [6,7,8,9]. In vitro studies have also reported that EGFR inhibitors effectively reduce the growth of OS cells [10,11]. However, as one previous study found that the EGFR inhibitor gefitinib (20 μM) was unable to reduce the viability of several OS cell lines [12], the role of EGFR in OS remains unresolved. Overall, a large-scale evaluation of EGFR mutations in OS is needed to determine the sensitivity of mutated EGFR to molecular targeting therapies [13]. Moreover, novel downstream targets of EGFR that act via different mechanisms should be identified to further improve the survival rates of OS patients. The binding of EGF to its receptor induces EGFR dimerization at the plasma membrane, which results in trans-autophosphorylation and activation of the tyrosine kinase domain [14,15], ultimately leading to modulation of a variety of intracellular pathways, including the MAPK and PI3K/AKT pathways [12].

Rho GTPases, small (21–25 kDa) GTP-binding proteins that belong to the Ras superfamily, play crucial roles in regulating cell migration, proliferation, polarity, apoptosis, cycle progression, adhesion, and motility [16,17,18,19]. EGF triggers cytoskeletal reorganization of actin microfilaments by regulating members of the Rho GTPase family, such as Rho A, Rac, and Cdc42 [20]. Although EGFR stimulation has been reported to lead to reorganization of the cytoskeleton, membrane ruffling and focal adhesion through Rho protein activation [21,22], the mechanistic details by which Rho proteins function in EGF-induced OS cell migration remain unclear.

In this study, we hypothesized that the migration of OS cells induced by EGF might be regulated by the Rho family of GTPases. As our preliminary experiments indicated that Rho A has an important role in this process, the purpose of this study was to determine the detailed mechanism of Rho A’s involvement. We confirmed that EGF (10 ng/mL) stimulated MG63 cell migration and explored expression of Rho A and its downstream targets (ROCK1, LIMK2, Cofilin1) in OS cells. We applied different inhibitors and determined their effects on OS cell migration. This newly discovered signaling cascade described herein mediates EGF-induced actin microfilament formation in MG63 cells and may provide a new target for preventing and treating OS.

## 2. Results

### 2.1. EGF Activates EGFR Expression in MG63 Cells and Promotes Cell Migration by Increasing Actin Stress Fiber Formation

**Figure 1:**
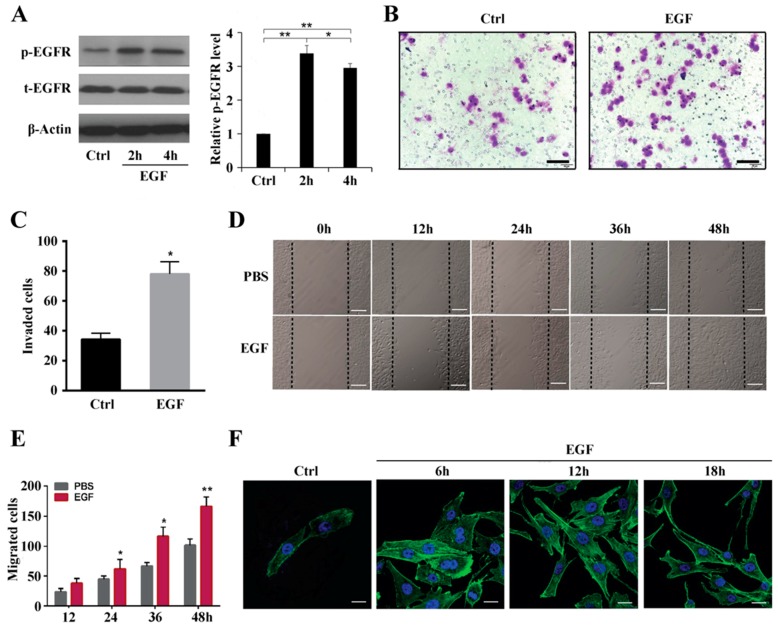
EGF promotes MG63 cell migration and stress fiber formation. (**A**) Expression of EGFR in MG63 cells after treatment with 10 ng/mL EGF for 2 and 4 h; (**B**) Transwell assay of MG63 cells treated with 10 ng/mL EGF; phosphate-buffered saline (PBS) treatment served as a control, scale bar = 200 μm; (**C**) Quantification of invaded MG63 cells treated with 10 ng/mL EGF; (**D**) Wound-healing assay of migrating MG63 cells induced with EGF for 12, 24, 36, and 48 h, scale bar = 200 μm; PBS treatment served as a control; (**E**) Quantification of migrating MG63 cells treated with EGF; (**F**) Immunofluorescent staining of stress fibers and DAPI staining of MG63 cells treated with PBS and EGF (10 ng/mL), scale bar= 50 μm. * *p* < 0.05, ** *p* < 0.01.

According to our preliminary results, a concentration of 10 ng/mL of EGF was deemed suitable for use in this study. Figure 1A shows that EGFR phosphorylation increased significantly after 2 h of treatment with EGF and reached a peak at 4 h, after which EGFR phosphorylation decreased. A transwell invasion assay was performed to explore the effect of EGF on MG63 cell migration. As shown in Figure 1B,C, 10 ng/mL EGF resulted in significantly increased invasion compared to the control group (*p* < 0.05). In addition, a wound-healing assay showed that MG63 cell migration was dramatically increased in the 10 ng/mL EGF-treated group compared to that in the control group at 12, 24, 36, and 48 h (*p* < 0.05). Furthermore, at 48 h, the scratch was completely filled with migrated MG63 cells (Figure 1D,E). Actin stress fibers have a fundamental role in providing force for several vital cellular processes, such as migration, cytokinesis, and morphogenesis. Therefore, we assessed the formation and distribution of actin stress fibers in MG63 cells treated with EGF. Figure 1F shows the increased distribution of actin stress fibers along the membrane after stimulation with EGF for 6 h in comparison to the control group. The distribution and formation of actin stress fibers around the nucleus in MG63 cells peaked from 12 to 18 h.

### 2.2. Rho A Is Involved in EGF-Induced Migration of MG63 Cells

**Figure 2:**
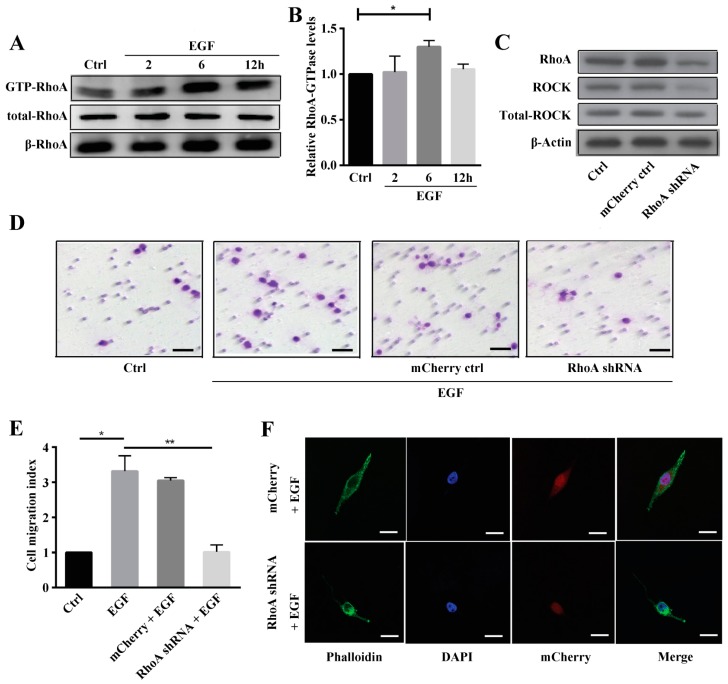
Rho A is involved in EGF-induced migration of MG63 cells. (**A**) Western blotting of GTP-Rho A expression in MG63 cells treated with EGF for 2, 4, and 6 h; (**B**) Quantification of the GTP-Rho A band obtained by western blotting; (**C**) Western blotting of Rho A and ROCK expression in MG63 cells treated with Rho A shRNA and the mCherry control; (**D**) Transwell assay of MG63 cells treated with PBS, 10 ng/mL EGF, 10 ng/mL EGF + mCherry, or 10 ng/mL EGF + Rho A shRNA, scale bar = 200 µm; (**E**) Quantification of invaded MG63 cells treated with PBS, 10 ng/mL EGF, 10 ng/mL EGF + mCherry, or 10 ng/mL EGF + Rho A shRNA; (**F**) Phalloidin staining of stress fibers and DAPI staining of MG63 cells induced with 10 ng/mL EGF + mCherry or 10 ng/mL EGF + Rho A shRNA, scale bar= 50 μm. * *p* < 0.05, ** *p* < 0.01.

Having established that actin stress fibers reorganize in MG63 cells following EGF treatment, we sought to determine the potential mechanism of this effect. A pull-down assay was performed to determine the role of Rho A in MG63 cell migration, and we found that GTP-bound Rho A increased significantly 6 to 12 h after treatment with EGF, as shown in Figure 2A,B. To further explore the role of Rho A in EGF-induced migration of MG63 cells, Rho A short hairpin RNA (shRNA) interference (approximately 80% efficiency (Figure S1)) was performed. Results of western blotting showed that after treatment with Rho A shRNA, the expression levels of Rho A and its downstream target ROCK were significantly reduced in MG63 cells compared to mCherry and control groups (Figure 2C). Next, we conducted a transwell assay and found that Rho A shRNA significantly reduced MG63 cell migration compared to control and mCherry groups (Figure 2D,E). Moreover, actin stress fiber reorganization was also inhibited in the Rho A shRNA-treated group (Figure 2F).

**Figure 3:**
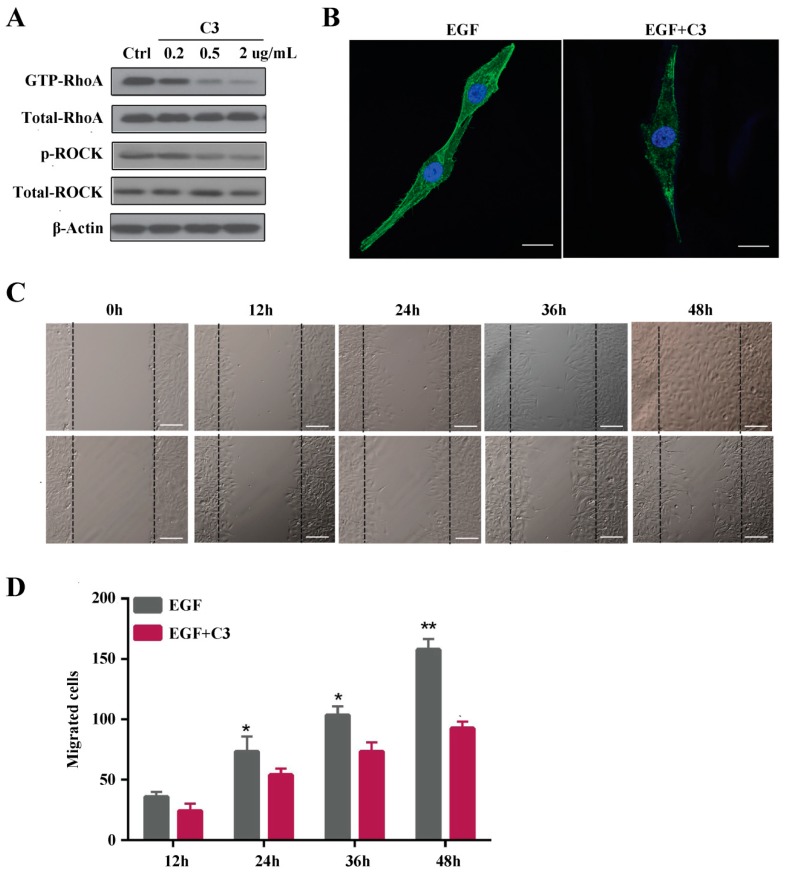
The Rho A inhibitor exoenzyme C3 inhibits EGF-induced MG63 cell invasion and stress fiber formation. (**A**) Western blotting of GTP-Rho A, Rho A, p-ROCK, and ROCK expression in MG63 cells pretreated with C3 at different concentrations followed by treatment with EGF; (**B**) Phalloidin staining of stress fibers and DAPI staining of MG63 cells with and without C3 pretreatment followed by the addition of EGF, , scale bar = 50 μm; (**C**) Results of a wound-healing assay in which MG63 cells with and without C3 pretreatment were treated with EGF for 12, 24, 36 or 48 h; (**D**) Quantification of migrated MG63 cells with and without C3 pretreatment followed by the addition of EGF for 12, 24, 36 or 48 h, , scale bar = 200 μm * *p* < 0.05, ** *p* < 0.01.

For further exploration of the role of Rho A in MG63 cell migration, the Rho inhibitor exoenzyme C3 was employed to specifically inhibit expression of Rho A and ROCK. Firstly, we confirmed that exoenzyme C3 was not affect the visibility of MG63 cells (Figure S2). Then as shown in Figure 3A, phosphorylation of Rho A and ROCK was inhibited by exoenzyme C3 in a concentration-dependent manner. Previously, we demonstrated that EGF increases actin stress fiber formation but that exoenzyme C3 treatment decreases it (Figure 3B). A wound-healing assay showed that MG63 cells treated with EGF gradually migrated to the scratch and completely filled it after 48 h. In comparison, MG63 cells treated with EGF plus exoenzyme C3 exhibited reduced migration at 24, 36 and 48 h compared to the group treated with EGF alone (Figure 3C,D).

### 2.3. ROCK Promotes MG63 Cell Migration and Stress Fiber Reorganization

**Figure 4:**
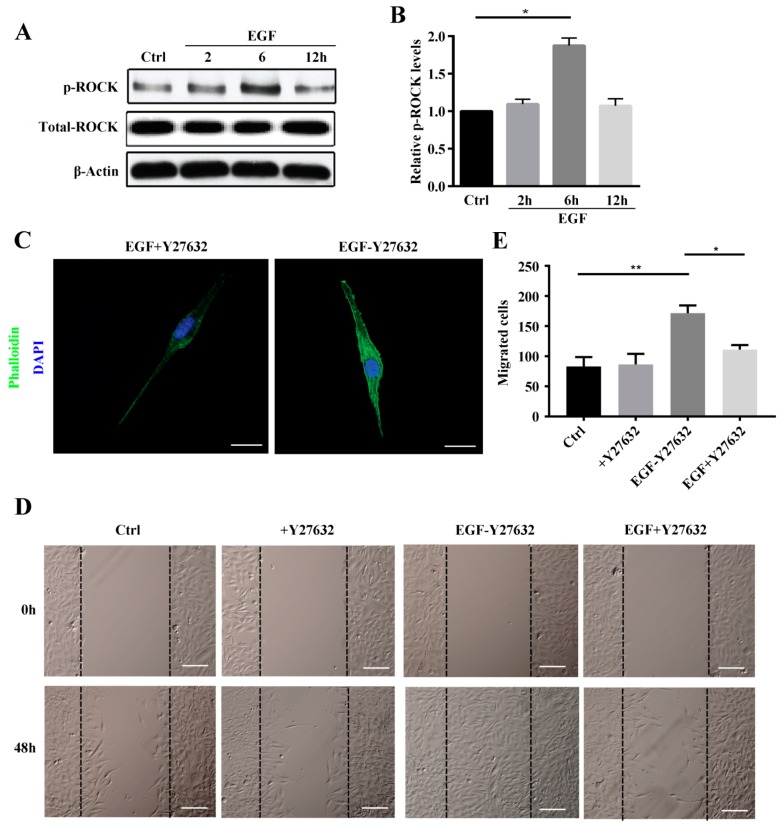
ROCK promotes EGF-induced stress fiber formation and cell migration in MG63 cells. (**A**) Western blotting of phosphorylated ROCK in MG63 cells treated with EGF at 2, 6, and 12 h; (**B**) Quantification of the ROCK band obtained by western blotting; (**C**) Phalloidin staining of stress fibers and DAPI staining of MG63 cells pretreated with Y27632 (10 μM) for 2 h followed by the addition of EGF, scale bar = 50 μm; (**D**) Wound-healing assay of MG63 cells treated with PBS, Y27632, EGF, or EGF + Y27632 for 48 h, scale bar = 200 μm; (**E**) Quantification of the migrated MG63 cells treated with PBS, Y27632, EGF, or EGF + Y27632 for 48 h. * *p* < 0.05, ** *p* < 0.01.

ROCK is a main downstream effector of Rho A, and its primary role involves regulating the shape and movement of cells by acting on the cytoskeleton. Thus, we examined whether Rho A promotes EGF-induced stress fiber organization and migration in MG63 cells via ROCK. MG63 cells were pretreated with the ROCK inhibitor Y27632 for 2 h and then treated with EGF. As shown in Figure 4A,B, we found that ROCK phosphorylation increased from 2 to 6 h, reaching a maximum at 6 h and returning to the baseline level at 12 h. Compared to the group not pretreated with Y27632, reorganization of stress fibers in the Y27632-pretreated group was significantly suppressed (Figure 4C). Furthermore, a wound-healing assay revealed no significant difference in MG63 cell migration between control and Y27632-treated groups. However, the number of migrated MG63 cells in the EGF-treated group was clearly increased compared to that in the control and Y27632-treated groups (*p* < 0.01). Interestingly, the number of migrated MG63 cells in the EGF + Y27632 group decreased compared to that in the group treated with EGF alone (*p* < 0.05).

### 2.4. ROCK Mediates MG63 Cell Migration via Activation of LIMK2/Cofilin1 Signaling

**Figure 5:**
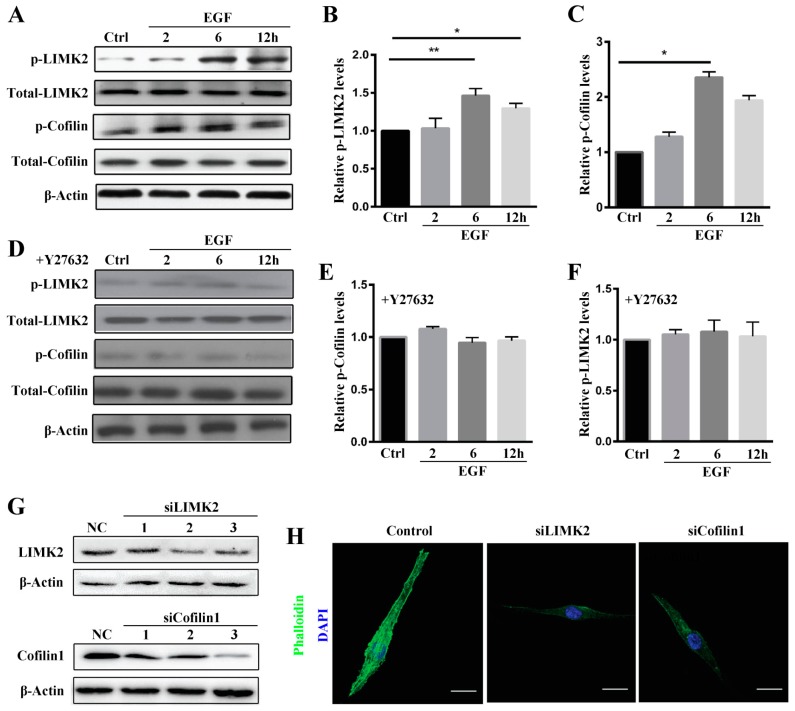
EGF activates LIMK2 and Cofilin expression in MG63 cells through ROCK. (**A**) Phosphorylation of LIMK2 and Cofilin in MG63 cells treated with 10 ng/mL EGF for 2, 6 and 12 h, as detected by western blotting. Quantification of LIMK2 (**B**) and Cofilin (**C**) phosphorylation in MG63 cells treated with 10 ng/mL EGF for 2, 6 and 12 h; (**D**) Phosphorylation of LIMK2 and Cofilin pretreated with Y27632 for 2 h followed by the addition of EGF, as detected by western blotting. Quantification of Cofilin (**E**) and LIMK2 (**F**) phosphorylation in MG63 cells pretreated with Y27632 for 2 h followed by the addition of EGF; (**G**) Expression of LIMK2 and Cofilin in MG63 cells treated with LIMK2 siRNA and Cofilin1 siRNA, as detected by western blotting. siR-Ribo™ served as the negative control (NC); (**H**) Phalloidin staining of stress fibers and DAPI staining of MG63 cells treated with LIMK2 and Cofilin1 siRNAs. siR-Ribo™ served as the negative control, scale bar = 50 μm. * *p* < 0.05, ** *p* < 0.01.

As Rho A activates downstream LIMK2 through ROCK, leading to inactivation and phosphorylation of the actin-depolymerizing factor Cofilin1 and subsequent cytoskeletal reorganization [23], we sought to identify whether ROCK mediates MG63 cell migration by activating LIMK2/Cofilin1 signaling. As shown in Figure 5A,B, LIMK2 phosphorylation significantly increased and reached a peak at 6 h and then declined at 12 h post-EGF stimulation. Cofilin1 phosphorylation showed a similar trend, increasing after 2 h and reaching a peak at 6 h (Figure 5A,C). Moreover, EGF-induced phosphorylation of LIMK2 and Cofilin1 was suppressed by the ROCK inhibitor‎ Y27632 (Figure 5D–F), suggesting that EGF induced LIMK2 activation and Cofilin1 activation through ROCK. We also examined whether LIMK2 and Cofilin1 regulate EGF-induced stress fiber formation and reorganization. To this end, we designed small interfering RNAs (siRNAs) for LIMK2 and Cofilin1 and found that their expression was clearly inhibited when MG63 cells were treated with the respective siRNA (Figure 5G). In addition, siLIMK2 and siCofilin1 inhibited stress fiber formation (Figure 5H), indicating that LIMK2 and Cofilin1 act downstream of Rho A/ROCK to regulate EGF-induced stress fiber formation in MG63 cells.

## 3. Discussion

EGF promotes OS migration through activation of EGFR, which positively correlates with TNM stage in OS. It is well known that most kinases activate their downstream effectors through phosphorylation; however, previous reports have shown that small molecule kinase inhibitors, such as Gefitinib and BIBW 2992, have little effect on OS development and no significant inhibitory effect on cell viability in vitro [24]. These results suggest that EGFR is not a central regulator of OS cell proliferation under in vitro cell culture conditions. Thus, it is imperative to identify the common molecular alterations that drive OS proliferation and migration, and there is an urgent need to identify effective targets that are downstream from EGFR in OS.

In this study, MG63 cell migration was induced with 10 ng/mL EGF, which is in agreement with a previous study demonstrating that EGF is a cytokine with multifunctional activity, even in the same cell type [25]. The results presented in Figure 1F indicate that actin stress fiber formation was induced by EGF at different time points. Thus, we hypothesized that Rho GTPases are involved in cell cytoskeleton formation and organization. EGFR activation reportedly leads to membrane reorganization, ruffling of the cytoskeleton and focal adhesions through activation of Rho GTPases [21], which are GTP-binding proteins involved in cell cytoskeleton formation and organization, as well as transcription, migration, and proliferation. Rho GTPases function as sensitive molecular switches existing either in an active GTP-bound form or in an inactive GDP-bound form, and the GTP hydrolytic activity of the former is responsible for their involvement in cytoskeleton rearrangement and cell motility [26]. Jian Han et al. reported that Rho A and Rho C play important roles in the EGF-mediated migration of trophoblast cells and that Rho A regulates this migration through F-actin cytoskeleton reorganization [27]. Taken together, we demonstrated that Rho A is clearly activated and in the GTP-bound form at 6 h after EGF stimulation. Additionally, we showed that Rho A plays a key role in MG63 cell migration when EGFR is overexpressed. To further clarify these findings, we inhibited GTP-Rho A with Rho A shRNA and the inhibitor C3. According to our results, Rho A inhibition suppresses MG63 cell migration and stress fiber formation. Furthermore, Rho A may be a downstream target of EGFR overexpression in OS. The downstream effectors of Rho A need to be examined and the signaling pathway dissected to reveal the mechanism of EGF-induced migration.

Rho-associated protein kinase (ROCK) belongs to the AGC (PKA/PKG/PKC) family of serine-threonine kinases, which are central regulators of the actin cytoskeleton that function downstream of the small GTPase Rho. ROCK signaling plays an essential role in a wide range of human diseases, and it has been recognized as a potential target and major modulator in the treatment of diseases involving abnormal cell motility, tumor cell invasion, and actin cytoskeleton organization [28,29]. Consistent with this, we found that ROCK inhibition reduced stress fiber formation. We also investigated ROCK downstream signaling to elucidate the mechanisms underlying ROCK participation in stress fiber formation. The data showed LIMK2 and Cofilin1 to be activated in MG63 cells after EGF treatment and that stress fiber formation was reduced by siRNA, which clearly demonstrates that the ROCK-LIMK2-Cofolin1 axis regulates stress fiber formation but does not inhibit cell migration.

In conclusion, our study shows that EGF (10 ng/mL) activates EGFR phosphorylation and stimulates MG63 cell migration, which can tailor the EGFR overexpression model. We herein provide evidence that MG63 cell migration and stress fiber formation occur through Rho A activation when EGFR is overexpressed. The downstream ROCK-LIMK2-Cofilin1 pathway also regulates the actin cytoskeleton formation in MG63 cells induced by EGF but not their migration. Our findings may have clinical implications that warrant further research using animal models to evaluate treatments for OS. This study has some limitations. First, we only used the MG63 cell line as a cell model, and additional OS cell lines, such as HOS, 143B, and Saos-2 cells, need to be explored. Second, primary OS cells from patients should be evaluated to assess the role of Rho A in regulating EGF-induced human OS cell migration. Third, evidence for the enhancement of migration and invasion after EGF treatment, as mediated through EGFR activation, is insufficient, and direct inhibition of EGFR activation by inhibitors or antibodies is required.

## 4. Materials and Methods

### 4.1. Materials and Reagents

The following antibodies served as primary antibodies: rabbit monoclonal antibodies against ROCK1 (Cell Signaling Technology, Danvers, MA, USA, #4035, 1:1000) and phosphorylated-ROCK (p-ROCK; Abcam, Cambridge, UK, #ab203273); rabbit polyclonal antibodies against LIMK2 (Cell Signaling Technology, Danvers, MA, USA, #3845) and phosphorylated-LIMK2 (p-Limk2; Sigma, St Louis, MO, USA, #SAB4300104); mouse monoclonal antibodies against Rho A (Santa Cruz Biotechnology, Dallas, TX, USA, #sc-418, 1:500). Antibodies against Cofilin (#5175) and p-Cofilin (#3311) were purchased from Cell Signaling Technology (Danvers, MA, USA).

EGF was obtained from Worthington Biochemical Corporation (Lakewood, NJ, USA). Regarding specific inhibitors, ROCK1 (Y-27632) was obtained from Selleck (Houston, TX, USA), and Rho Inhibitor I (CT04-A) was purchased from Cytoskeleton (Danvers, MA, USA). Whole Cell Lysis Protein Extraction Kit was purchased from Nanjing keyGEN BioTECH, Ltd. (Nanjing, China). Actin-Tracker Green was obtained from Shanghai Beyotime, Ltd. (Shanghai, China). UltraCruz^®^ Hard-set Mounting Medium was purchased from Santa Cruz Biotechnology (TX, USA, #sc-359850).

### 4.2. Cell Culture

MG63 cells were obtained from the Department of Histology and Embryology, Southern Medical University (Guangzhou, China). MG63 cells were cultured in Dulbecco’s Modified Eagle’s Medium (DMEM, Corning, Herndon, VA, USA) containing 10% fetal bovine serum (FBS, SeraBest, Aidenbach, Bavaria, Germany), 100 U/mL penicillin and 100 μg/mL streptomycin (Gibco, Grand Island, NY, USA) at 37 °C in a humidified atmosphere of 5% CO_2_. Unless otherwise noted, this medium was used as the standard medium. In some experiments, MG63 cells were pretreated with the ROCK1 inhibitor Y-27632 (10 mM; Selleck, TX, USA). EGF was added to the culture medium at the indicated time points (2, 6, and 12 h).

### 4.3. Pull Down Assay

Activity of Rho GTPases was assessed in OS cells using a pull-down assay for GTP-bound Rho according to the manufacturer’s protocol (Rho Assay Reagent (Rhotekin RBD, Agarose), Millipore, Burlington, MA, USA, cat. #14-383). GTP-bound Rho A was precipitated from cell lysates with PAK-GST Protein Beads ((human p21 activated kinase PBD) Cat. #PAK02). Briefly, MG63 cells were starved in serum-free medium for 24 h, rinsed with ice-cold Tris-buffered saline (TBS) and lysed in 600 µL of RIPA for 10 min. An aliquot (120 µg, 600 µL) of the extract was placed into two experimental tubes; GDP (1/100th of the aliquot volume) was then added to one tube, whereas GTPγS (1/100th of the aliquot volume) was added to the other tube. The tubes were incubated at room temperature for 15 min. The reaction was stopped by adding 1/10th the volume of stop buffer to each tube (final conc. 60 mM MgCl_2_). PAK-GST protein beads were resuspended, and 20 µg (20 µL) of the protein-bound beads was added to each reaction tube. The tubes were gently rotated at 4 °C for 1 h and then centrifuged at 5200× *g* at 4 °C for 1 min. The supernatant was removed and the beads were washed twice with 500 µL of wash buffer. The beads were pelleted and suspended in 25 µL of sodium dodecyl sulfate (SDS) sample buffer. The bound proteins were boiled in sample buffer at 99 °C for 10 min and then analyzed by western blotting to detect GTP-Rho A.

### 4.4. Lentiviral shRNA and Transduction In Vitro

Knockdown of Rho A was achieved via lentiviral transduction of human Rho A shRNA (sc-29471-V; Santa Cruz Biotechnology). According to the manufacturer’s instructions, scrambled shRNA (SC-108080) and the mCherry control (LPP-MCHR-LV105-025-C) were also included. The lentiviral particle solution was added to MG63 cells in fresh DMEM containing 5 mg/mL polybrene. The medium was changed after 12 h of infection. After successive 12-h intervals, the medium was changed to fresh DMEM, and the cells were cultured for 72 to 96 h. After transduction, quantitative reverse transcription-polymerase chain reaction (qRT-PCR) analysis was employed to determine the knockdown efficiency.

### 4.5. Real-Time Quantitative PCR

qRT-PCR of total RNA from MG63 cells was extracted using TRIzol reagent (Invitrogen) according to the manufacturer’s instructions, and then 1 μg of RNA was reverse transcribed to generate cDNA using superscript reverse transcriptase (Invitrogen). qRT-PCR was carried out using a Real-time Quantitative PCR Detecting System. The primer sequences were as follows: Rho A shRNA (sc-29471-PR; Santa Cruz Biotechnology); GAPDH forward: 5′-TGACTTCAACAGCGACACCCA-3′; GAPDH reverse: 5′-CACCCTGTTGCTGTAGCCAAA-3′. The relative amounts of each transcript were calculated using the comparative *C*t (2^−ΔΔ*C*t^) method.

### 4.6. siRNA and Transfection In Vitro

The sequences of the human Cofilin1 and LIMK2 genes were obtained from GenBank (gene ID 1072 and 3985). Sequence-specific siRNAs targeting Cofilin1 and LIMK2 (Cofilin1 siRNA (5′-GGATCAAGCATGAATTGCA-3′) and LIMK2 siRNA (5′-GCTGCAAGGTGATCATTGA-3′)) were designed and synthesized by RiboBio Co., Ltd. (Guangzhou, China); a negative control siRNA (NC) was also designed by RiboBio Co., Ltd. All chemical transfections were performed using 60 nM of each siRNA and Lipofectamine™ 2000 (Invitrogen, Carlsbad, CA, USA), as described by the manufacturer. After 6 h at 37 °C, DMEM was replaced with complete growth medium (DMEM with 20% FBS). Cells not transfected with siRNA were used as an untreated control. Transcription expression was evaluated by western blotting.

### 4.7. Wound-Healing Assay

MG63 cells were seeded in 6-well plates at a density of 10^6^ cells/well and allowed to reach adequate confluence over one day in 2.5 mL of DMEM supplemented with 10% FBS. Thereafter, the medium was removed and replaced with serum-free medium. On the next day (24 h), sterile 200 μL pipette tips were used to scrape the monolayer of MG63 cells across each well, creating a cell-free area. The cell monolayer was washed gently with phosphate-buffered saline (PBS) to remove detached cells and cell debris. At various time points (12, 24, 36, and 48 h), images were captured using an inverted microscope (Olympus, Tokyo, Japan) at 20× magnification to quantify the closure of the scratch. Differences between the wound area at time 0 and the other four time points were determined. The scratch area was measured using ImageJ software (National Institutes of Health, Bethesda, MD, USA).

### 4.8. Transwell Migration Assay

MG63 cells transfected with Rho A shRNA and the inhibitor Y27632 and negative control cells were starved overnight in serum-free medium. EGF (10 ng/mL) was added to the cells for 24 h before treating with trypsin. Cells (2 × 10^5^) in 400 µL of medium were added to the top chamber of 24-well transwell plates (8 µm pore size, Corning, NY, USA). The bottom chambers were filled with 600 µL of medium containing EGF (10 ng/mL). Transwell cultures without EGF were used as a control. After 24 h under standard culture conditions, non-migrated cells on the upper side of the membrane were removed with a cotton swab. The cells at the bottom of the filter were fixed with Immunol Staining Fix Solution (Beyotime, Shanghai, China) and stained with 0.1% crystal violet at room temperature. After air-drying, the membrane was mounted on a glass slide and examined under a microscope. All experiments were repeated at least 3 times.

### 4.9. Fluorescent Staining of the Cytoskeleton

MG63 cells were washed three times with PBS and fixed with Immunol Staining Fix Solution (Beyotime) for 10 min, after which time they were washed with Immunol Staining Wash Buffer (Beyotime) 3 times for 5 min each. Actin-Tracker Green was diluted in Immunol Fluorescence Staining Secondary Antibody Dilution Buffer (Beyotime) and incubated with the cells for 40 min in the dark, after which they were washed with Immunol Staining Wash Buffer 3 times for 5 min each. Finally, nuclei were stained with UltraCruz^®^ Hard-set Mounting Medium including 4′,6-diamidino-2-phenylindole (DAPI; sc-359850, Santa Cruz Biotechnology). Images were recorded using a Zeiss confocal laser-scanning microscope equipped with a Plan-Apochromat 60× oil-immersion objective lens (Zeiss LSM 880 Confocal Laser Scanning unit, Carl Zeiss AG, Oberkochen, Germany). Image processing, combining and analysis were performed using the ZEN software package (Carl Zeiss MicroImaging GmbH, Jena, Germany).

### 4.10. Western Blotting

To extract proteins, cultured MG63 cells were collected and lysed in 1X RIPA buffer according to the instructions. The protein content was determined using a BCA Protein Assay Kit (Beyotime, Shanghai, China) using BSA (bovine serum albumin) as the standard. Samples were boiled for 5 min at 99 °C and separated by 10% SDS-polyacrylamide gel electrophoresis (PAGE) using running buffer (190 mM glycine, 25 mM Tris, 0.1% SDS) and then transferred to polyvinylidene fluoride (PVDF) membranes (Millipore, MA) using transfer buffer (190 mM glycine, 25 mM Tris, 30% methanol). The membranes were blocked for 2 h in 5% milk powder in TBS-Tween-20 (TBST) at room temperature, incubated overnight at 4 °C with the primary antibody diluted 1:1000 in 5% BSA. After washing three times with TBST, the membranes were incubated with a secondary antibody for 1 h at room temperature. Finally, the membranes were stained with ECL Western Blotting Substrate (Thermo, Waltham, MA, USA), scanned, and stored using a gel imaging system. The protein bands were analyzed using Image Lab software. Images shown in figures are representative of 3 independent experiments.

### 4.11. Statistical Analyses

All experiments were repeated three times. SPSS software (version 13.0, SPSS Inc., Chicago, IL, USA) was used for all statistical analyses, and the data are expressed as the mean ± SD. Statistical differences between groups were determined by one-way analysis of variance (ANOVA). A *p*-value < 0.05 was considered significant, and the significance level was defined as * *p* < 0.05, ** *p* < 0.01.

## Supplementary Materials

Supplementary materials can be found at http://www.mdpi.com/1422-0067/19/5/1437/s1.
