# Supplementary material for: Rho A Regulates Epidermal Growth Factor-Induced Human Osteosarcoma MG63 Cell Migration
Source: Int J Mol Sci. 2018 May 11;19(5):1437. doi: 10.3390/ijms19051437 (PMC5983621; doi:10.3390/ijms19051437)
Supplement: Supplementary file 1 [file ijms-19-01437-s001.pdf]

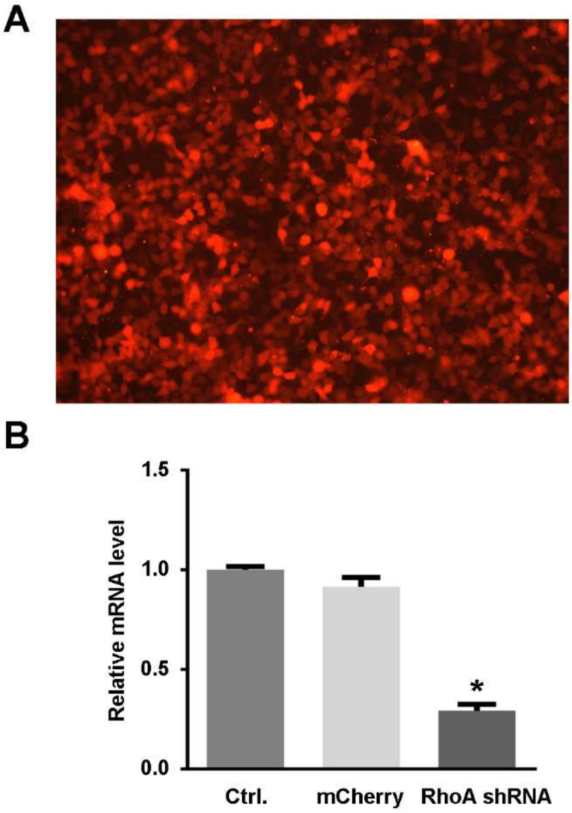

Figure S1. The demonstration of Rho A shRNA transfection efficiency.

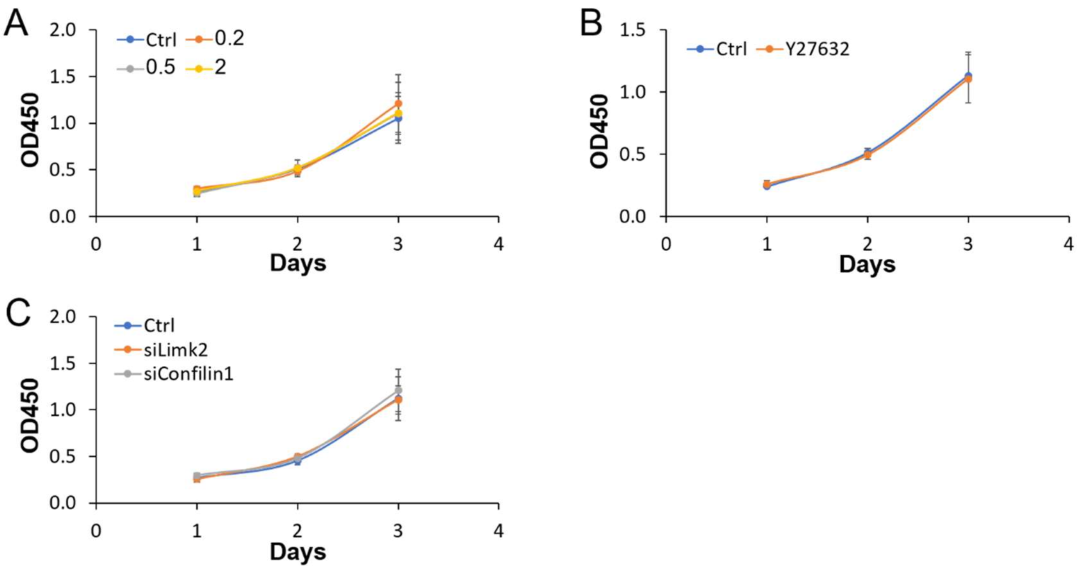

Figure S2. (A) The proliferation of MG63 cells treated with 0.2, 0.5, 2 mg/ml C3, cells without any treatment as control. (B)The proliferation of MG63 cells treated with or without Y27632. (C) The proliferation of MG63 cells treated with siRNA-Limk2 and siRNA-Confilin1, cells without any treatment as control.
